# Age-associated bimodal transcriptional drift reduces intergenic disparities in transcription

**DOI:** 10.18632/aging.101428

**Published:** 2018-04-27

**Authors:** Byungkuk Min, Myungsun Park, Kyuheum Jeon, Jung Sun Park, Hyemyung Seo, Sangkyun Jeong, Yong-Kook Kang

**Affiliations:** 1 Development and Differentiation Research Center, Korea Research Institute of Bioscience and Biotechnology (KRIBB), Yuseong-gu, Daejeon 34141, South Korea; 2 Department of Molecular and Life Sciences, Hanyang University, Sangnok-gu, Ansan, Gyeonggi-do 15588, South Korea; 3 Mibyeong Research Center, Korea Institute of Oriental Medicine (KIOM), Daejeon305-811, Korea; * Equal contribution

**Keywords:** aging, gene expression, T cells, Huntington’s disease, multiplex PCR, and transcription

## Abstract

This study addressed the question of how well the quantitative transcriptome structure established in early life is maintained and how consistently it appears with increasing age, and if there is age-associated alteration of gene expression (A_3_GE), how much influence the Huntington’s disease (HD) genotype exerts on it. We examined 285 exonic sequences of 175 genes using targeted PCR sequencing in skeletal muscle, brain, and splenic CD4+ T cells of wild-type and HD mice. In contrast to the muscle and brain, T cells exhibited large A_3_GE, suggesting a strong contribution to functional decline of the organism. This A_3_GE was markedly intensified in age-matched HD T cells, which exhibited accelerated aging as determined by reduced telomere length. Regression analysis suggested that gene expression levels change at a rate of approximately 3% per month with age. We found a bimodal relationship in A_3_GE in T cells in that weakly expressed genes in young mice were increasingly transcribed in older animals whereas highly expressed genes in the young were decreasingly expressed with age. This bimodal transcriptional drift in the T cell transcriptome data causes the differences in transcription rate between genes to progressively reduce with age.

## Introduction

Aging is a multifactorial process during which molecular alterations such as genetic and epigenetic mutations accumulate, resulting in decrepitude, frailty, and untimely death. Aging is of significant importance for human health because it is the primary risk factor for a variety of diseases including cancer, metabolic disorders, and neurodegenerative diseases [1–4]. Great strides have recently been made by studies that have led to a systematic categorization of the molecular hallmarks of aging [5]. These individual traits interact with the transcriptional network, which directly influences the transcriptomic profile. Therefore, the exploration of age-associated features of gene expression is fundamental for elucidating the mechanisms underlying the deteriorated cellular functions observed during aging and in age-related disorders. Transcriptomic signatures of aging have indeed been reported for a number of species and tissues (for review, see [6]). These massive scale studies have mostly aimed at detecting either tissue specific or tissue independent aging marker genes that show statistically significant age related changes in expression levels, but a few have been interested in identifying the patterns of transcriptional drift of individual genes and the elements that decide the pattern.

In this study, we aimed at quantifying the differences in expression levels of a collection of epi-driver genes in young and old mouse samples. These epi-driver genes encode proteins which comprise the epigenome and are directly involved in a variety of epigenetic mechanisms [7]. We utilized the method of spiking-in a neighbor genome for competitive PCR-amplicon sequencing (SiNG-PCRseq) [7,8]. One of the benefits of SiNG-PCRseq is the high level of accuracy with which the quantities of target transcripts can be measured by virtue of the spike-in rat genomic DNA. For comparison, we also examined Huntington’s disease (HD) model mice in which we observed an accelerated aging phenotype in the previous study [7]. HD is considered as mainly a central nervous system (CNS) disorder but ubiquitous expression of both normal and mutant Huntingtin (HTT) in the whole body [9] has attracted attention toward peripheral dysfunctions the study of which has increased knowledge of HD etiology and has aided the identification of new biomarkers [10,11]. As evidenced by the flourishing number of transcriptional and proteomic studies conducted on HD blood [12,13]. On examination of the comparative transcriptome analyses of tissue samples of different ages and genotypes, we discovered that the expression levels of epi-driver genes were altered with age and this age-associated alteration of gene expression (A_3_GE) occurred progressively and, in particular, with the T cells displaying an interesting pattern of transcriptional drift.

## RESULTS

### Measurement of gene expression levels in young and old samples using SiNG-PCRseq

For gene expression analysis, we used a set of primer pairs that can amplify 285 exonic amplicon sequences of 175 epi-driver genes [7]. Using SiNG-PCRseq, we determined the amount of target transcript levels in the skeletal muscle, brain (striatum), and splenic CD4+ T cells collected from wild type young (wy, 2 months old) and old (wo, 16–19 months old) mice and age-matched HD mice (hy and ho, respectively). In running multiplexed SiNG-PCR, rat genomic DNA (gDNA) was used as a spike-in for accurate quantitation of mouse cDNA sequences relative to matched rat gDNA sequences [7]. Each of the primer pairs was designed to carry one (or >2 less frequently) nucleotide variation between the DNAs of different origin species to best achieve their equal amplification and to set distinguishable flags between the cDNA-derived reads and the rat gDNA reads after deep sequencing. Splenic CD4+ T cells were pooled from three or four mice and split into four replicates before use in multiplex PCR. Brain and muscle samples were analyzed from individual animals. We obtained 6.6 × 10^6^ (muscle), 8.5 × 10^6^ (brain), and 29.6 × 10^6^ (T cells) reads on average per index after sequencing about 80% of which were mapped to our amplicon-built reference (Supplementary Data File 1).

The expression level of each exonic sequence was measured by calculating the M/R ratio, the ratio of the mouse read counts relative to the rat’s counts (Supplementary Data File 1). Principal component analysis (PCA; Supplementary Fig. S1) showed that the muscle samples did not differ much, whereas brain tissue showed a moderate distinction between ages and also between the genotypes. The young-old difference was the most prominent in T cells. The young T cells of wild-type and HD mice grouped together but with age the two genotypes diverged greatly and became distinct from each other.

### Age-associated transcriptional alteration in splenic T lymphocytes

**Figure 1:**
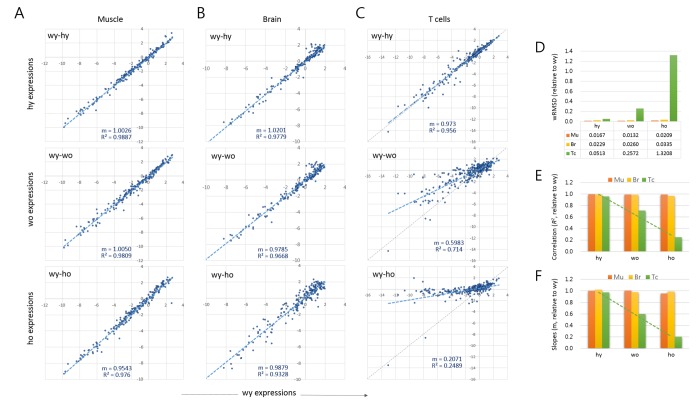
**Age-associated changes in quantitative transcriptome structure of splenic T cells.** (**A**-**C**) Scatter plots comparing the epi-driver gene expression levels between wild-type young (wy) and HD young (hy) samples (wy-hy), between wild-type young (wy) versus wild-type old (wo) samples (wy-wo), and between wild-type young versus HD old (ho) samples (wy-ho) in skeletal muscle (**A**), brain tissue (**B**), and splenic CD4+ T cells (**C**). Blue and gray lines denote a regression curve and the reference slope (m = 1.0), respectively. (**D**) Weighted root mean square deviation (wRMSD) analysis for assessment of the extent of difference in gene expression level between young and old cells. (**E**-**F**) Comparison of the correlations (coefficient of determination, R^2^; (**D**) and the slopes (**E**) of the scatter plots. Mu, muscle; Br, brain; Tc, T cells. Dotted green lines indicate trend lines for T cell samples.

We used a scatter plot to compare the expression levels between young versus old samples. Each of the tissue samples showed a high similarity between wild-type young versus HD young (wy-hy; Fig. 1A-C). Therefore, we took wy as the reference for the age-related changes occurring in both wild-type and HD samples. In muscles, the young to old comparisons (wy-wo and wy-ho) showed no substantial differences, as shown by their strong correlations (*R^2^* > 0.976). In the brain, however, a relatively large proportion of genes deviated to a degree from the regression curve for old HD (wy-ho, *R^2^* = 0.933). These alterations of epi-driver gene expression levels are potentially related to pathogenic events in the old HD brain and appeared to occur randomly as judged by the almost undisturbed plot slope (m = 0.9879; Fig. 1B).

The age-related changes to epi-driver gene expression were distinctive in the splenic T cells, as shown by the markedly lower plot slopes compared to muscle and brain (Fig. 1C**).** The young-old difference in expression levels was quantified by calculating the weighted root mean square deviation (wRMSD) of old samples from young samples [14–16]. The deviation was the highest in old HD T cells (wy-ho) and next from the old wild-type T cells (wy-wo), with the wRMSD values 79.3 and 15.4 fold larger, respectively, compared with those of young muscles (Fig. 1D). In the T cell samples, the plot correlations and slopes also decreased in the order hy, wo, and ho (Fig. 1E and 1F, respectively). While the wRMSD, correlation coefficients, and slopes could all be used to quantify gene expression differences, the former two cannot express the change of direction (increase or decrease) in gene expression; thus, we chose the slope (m) as the tool to express the age-associated alteration of gene expression, A_3_GE, which we defined as

$$\sqrt{{\left(1-m\right)}^{2}}$$.

### Alteration in the quantitative transcriptome structure as a function of age in T cells

**Figure 2:**
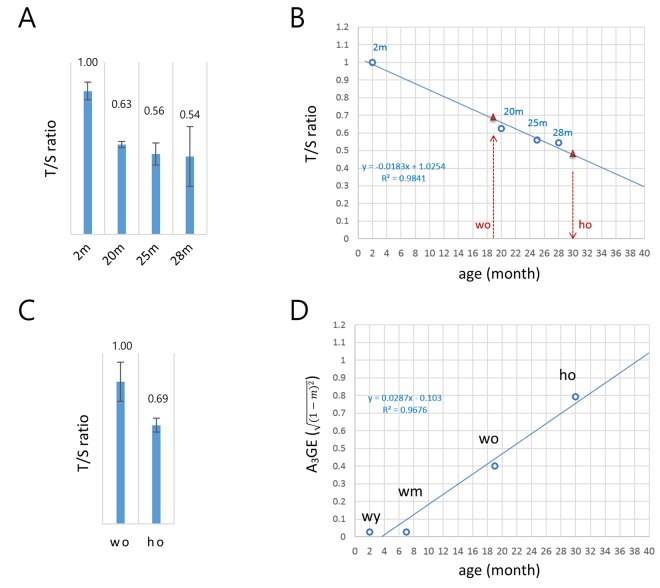
**A model for the alteration of gene expression levels as a function of age in T cells.** (**A**) Determination of the copy-number ratio (T/S ratio) of the telomeric sequence to a single-copy gene sequence (*S6B4*) in splenic CD4+ T cells of wild-type mice with different ages (2, 20, 25, and 28 months). Error bars, standard deviation. (**B**) Determination of the biological age of the ho T cells. Regression curve (dotted blue line) was derived by the T/S ratios of wild-type T cells in **A**. The T/S ratio of the 19 month old wo sample corresponds to approximately 0.68 (left red triangle). The relative T/S ratio of the ho T cells was 69% of the wo T/S ratio; the T/S ratio of the ho is 0.47 (0.68 x 0.69), which corresponds to about 30 months of age in wild-type mice (right red triangle). (**C**) Relative T/S ratio of the old HD (ho) T cells to the old wild-type (wo) T cells. In **A** and **B**, quantitative real-time PCR was performed to obtain C_t_ values for the telomere repeats and *S6B4* sequence before calculating the copy-number difference. Error bars, standard deviation. (**D**) A regression model of the age-associated alteration of gene expression (A_3_GE defined as $\sqrt{{\left(1-m\right)}^{2}}$, where m is a slope) in splenic T cells. A_3_GE increases at a rate of approximately 2.87% per month on average in wild-type mice.

Figure 1 demonstrated that A_3_GE is clearly identifiable in the CD4+ T cells. The exacerbated A_3_GE in old HD T cells might result from accelerated aging in HD mice. To test whether aging progresses more rapidly in HD T cells, we assessed the biological ages of old T cells by measuring their telomere lengths [17,18]. The copy number ratio (T/S ratio) of the telomeric sequence relative to a single-copy gene sequence (3*6B4*) [19,20] was first determined in T cells from 2, 20, 25, and 28 month old wild-type mice. The T/S ratio was found to decrease with age (Fig. 2A) at a rate of 1.83% per month, and there was a strong inverse correlation with age (*R^2^* = 0.9841; Fig. 2B).

Comparing old HD (16-19 months) and old wild-type (19 months) T cells, the T/S value of the former was 69% of the latter (Fig. 2C). This result provides evidence for accelerated aging in HD T cells. As shown in Fig. 2B, the relative T/S value of 19 month old, wild-type T cells corresponded to 0.68 on the linear regression if the T/S ratio of two month old T cells was set to 1.0. Hence, the T/S value of old HD T cells would be 0.47 (0.68 x 69%), which implies that they were comparable to 30 month old wild-type T cells. Hence, in terms of telomere length, the 16–19 month old HD T cells aged approximately 1.5-fold more than the age-matched wild-type T cells. Meanwhile, seven month old wild-type (wm) T cells had gene expression levels comparable to the young T cells, exhibiting no noticeable sign of A_3_GE (Supplementary Fig. S2). This suggests that A_3_GE does not start at the beginning of life but is somehow initiated during middle of life. Fig. 2D shows that A_3_GE values of wild-type T cells of different ages and age-adjusted old HD T cells exhibit a linear relationship with age (*R^2^* = 0.9676). In this regression model, the regression coefficient was 0.0287, implying that A_3_GE increases at a rate of 2.87% per month on average and reaches 50% at approximately 21 months of age in wild-type mice.

### Categorical analysis of epi-driver genes for age-associated alteration of gene expression

**Figure 3:**
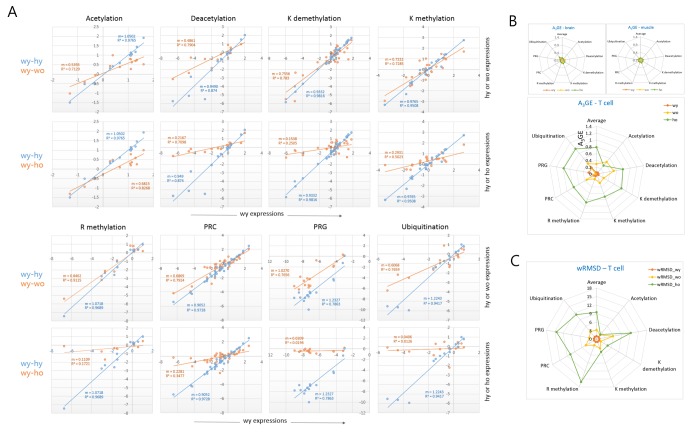
**Comparison of A_3_GE in different epi-driver gene categories between old wild-type and HD T cells.** (**A**) Difference in the extent of A_3_GE between wo and ho T cells. Two scatter plots which compare expression levels between young (wy-hy) and old (wy-wo; blue) and between young and HD old (wy-ho; orange) are merged. The slope (m) of the regression curve and the correlation value (*R*^2^, coefficient of determination) are shown. Each slope represents the extent of A_3_GE in the corresponding T cells. PRC, Polycomb-repressive complex; PRG, PRC-regulated genes. (**B**-**C**) Comparisons of the levels of A_3_GE (**B**) and weighted root mean square deviation (wRSMD) (**C**) in individual gene expression categories in young (wy and hy) and old (wo and ho) T cells. A_3_GE was minimal in muscle and brain samples which are displayed in the smaller plots. A_3_GE, age-associated alteration of gene expression.

We classified the epi-driver genes into eight different categories according to their roles in the modification of chromatin and compared their expression levels between young and old T cells. In all categories, a decrease of the plot slope was consistently observed in the old wild-type T cells, and it was even more severe in old HD T cells (Fig. 3A). When the A_3_GE in individual epi-driver categories was compared a relatively uniform A_3_GE was observed across the categories (Fig. 3B). An exception was the acetylation category in which the HD genotype dependent A_3_GE intensification was undetectable. This probably indicates transcriptional stability of acetylation category genes with age. In the muscle and brain samples, however, A_3_GE was not observed (see also Supplementary Fig. S3A and S3B). The slope and A_3_GE values for individual categories are summarized in the Supplementary Fig. S4. The extent of A_3_GE was so marked that the correlation among the genes was completely lost in some categories including the arginine-methylation (*R^2^* = 0.172), PRG (0.019), and ubiquitination categories (0.013; Fig. 3A). wRMSD calculations showed that the acetylation category was least affected whereas the PRC-regulated gene (PRG) category deviated with age the most (Fig. 3C), which is similar to the result of A_3_GE measurements in Fig. 3B.

In addition, we examined a further gene category that consisted of aging-related genes [7]. It showed a similar A_3_GE in the T cells (Supplementary Fig. S5A), indicating that A_3_GE was not restricted to the epi-driver genes. Another group of genes, which were selected from the literature for their relatively constant expression in various tissues, showed only a weak A_3_GE in T cells (Supplementary Fig. S5B). These observations suggest that the extent of A_3_GE is determined by the intrinsic stringency of gene expression regulation. We also examined MACS-purified splenic macrophages from old wild-type mice. SiNG-PCRseq results showed that epi-driver gene expression levels did not noticeably change with age in these cells (m = 0.988 and *R^2^* = 0.907; Supplementary Fig. S6), which refutes the possibility that A_3_GE affects all blood cells similarly.

### Transcriptional drift − Age-related reduction of gene-to-gene disparity of transcriptional activity

**Figure 4:**
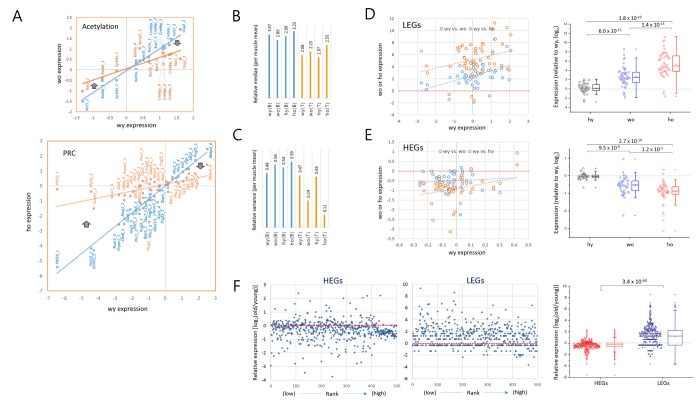
**Opposite transcriptional drift of genes with weak and strong transcriptional strengths.** (**A**) Representative pattern of an age-related expression changes in the acetylation (upper) and PRC (Polycomb repressive complex; lower) category genes. The expression levels of individual epi-driver genes in young T cells (blue) are paired with corresponding genes in old T cells (orange). The thick arrows denote the direction of expression-level change in low expressing genes (left) and high expressing genes (right) with age. (**B**-**C**) Measurement of the median (**B**) and variance (**C**) of gene expression levels in the brain (B) and T cells (T) relative to those in the muscle samples. (**D**-**E**) Opposite directions of age-associated alteration of gene expression (A_3_GE) between the lowly (LEGs, *n* = 50; D) and highly expressed genes (HEGs, *n* = 50); (**E**). Two scatter plots compare expression levels between young (wy-hy) and old (wy-wo; blue) and between young and HD old (wy-ho; orange) are merged. Box plots on the right show the opposing directions of change and also the statistical significance of the A_3_GE differences between the samples. (**F**) A_3_GE in the transcriptomes of CD4+ T cells. RNA-seq was performed using mRNAs of MACS-purified splenic CD4+ T cells pooled from three different mice of two or 20 months of age. Five hundred top and 500 bottom ranked genes were selected, HEGs (FPKM values ranging from 144.1 to 7,444.7) and LEGs (from 0.0041 - 0.035) respectively, after transcriptomes were sorted by expression level for FPKM levels of young T cells. Expression levels of old wild-type T cells relative to young were calculated using a log_2_ scale. P-values in D-F, paired sample *t*-test.

Another interesting feature of A_3_GE was that, as shown by the lowered plot slopes in Fig. 3A, those genes that were weakly expressed in young samples tended to be increasingly transcribed in old samples, whereas those genes which were relatively highly expressed in young cells tended to decline in old cells. Representative trends are presented for the acetylation and PRC category plots (Fig. 4A). The median values of relative (M/R) gene expression levels for all of the epi-driver genes ranged from 1.97 to 2.55 and did not greatly differ among the four T cell sample groups (Fig. 4B). However, interestingly, the variance of gene expression levels of them decreased to a half in the old wild-type T cells and to a quarter in old HD T cells compared to the young cells (Fig. 4C). In contrast, the muscle and brain samples showed no apparent change in variances. The reduced variance in old T cells demonstrates that the gene expression system in old T cells tends to narrow the differences in expression level between strongly and weakly transcribed genes.

In order to validate the general nature of this convergence of expression, we examined A_3_GE in two groups of genes, top ranked (highly expressed genes, HEGs; *n*=50) and bottom ranked (lowly expressed genes, LEGs; *n* = 50) determined after sorting the genes of the young wild-type T cells by expression level. We observed that the LEGs mostly showed increased levels in the old T cells and, conversely, the HEGs showed decreased levels (Fig. 4D and 4E, respectively). This tendency to plateau was far more obvious for the LEGs; the fold change in expression level was 2.7 (wo) to 5.3 (ho) on a log_2_ scale in the LEGs while it was 0.5 (wo) to 0.9 (ho) in the HEGs. Box plots demonstrate the opposing directions of expression change and the significant differences between LEGs and HEGs. The increasing transcription of LEGs in old T cells was not due to RNA polymerase II activity was decreased in old T cells (Supplementary Fig. S7), agreeing with a previous report of the downregulation of RNA splicing and processing genes in old cells [21].

We further examined the whole transcripts for A_3_GE in old wild-type mice. RNA-seq was performed using mRNAs from splenic CD4+ T cells and peripheral blood mononuclear cells (PBMCs). Fifty-four million reads on average per sample (82.5%) were uniquely mapped onto the mm10 reference (Supplementary Data File 1). Comparison of the expression levels of LEGs and HEGs (*n* = 500 each) revealed a similar trend of transcriptional drift between two and 20 month old mice and displaying a significant difference between the HEGs and LEGs (*p* = 3.4 x 10^-60^, paired sample *t*-test; Fig. 4F). In contrast to the CD4+ T cells, PBMCs showed no such a change for HEGs and only a slight increase in expression level for LEGs (Supplementary Fig. S8). These data indicate that this pattern of transcriptional drift, of all the cell types tested, is unique to CD4+ T cells.

## DISCUSSION

As demonstrated in Fig. 1C, old T cells unambiguously showed A_3_GE that was markedly aggravated in the HD genotype. In support of this result, previous studies have observed that there was a substantial change in mRNA expression in the blood of HD patients [13,22–24]. Moreover, the T cell A_3_GE was distinguished by the opposing patterns of transcriptional drift – the upregulation of the low expression level genes and the downregulation of the high expression level genes (Fig. 4A). This convergence reduces the difference in expression levels between low and high transcription activity genes, suggesting a progressive loss of discrimination in the regulation of gene expression with age. Given that aging is accompanied by changes in chromatin structure [6,25], this bidirectional A_3_GE may be explained by the age-associated perturbation of epigenomic structure such as loss of nucleosome occupancy and subsequent increase of chromatin opening [26,27]. These changes increase transcriptional noises among barely expressed genes, probably in a stochastic fashion [28], which consequently appropriate cellular resources for transcription. This individually tiny (at the level of individual genes), but collectively immense, pan-genome increase in transcription may cause a scarcity of nuclear resource for the transcription of major expressing genes. This scenario assumes a re-distribution of transcriptional resource from the majorly expressed genes to the minimally expressed genes with age.

As described above, A_3_GE is likely to be accompanied by perturbation of the epigenomic structure, particularly genomic DNA methylation. Age-associated changes in DNA methylation occurs as a combination of two contrasting effects: a global decrease but local increases of DNA methylation (for review, see [29]). These bimodal DNA methylation changes occur progressively as a function of age, the so called ‘epigenetic drift’ [30] and some local, specific methylation changes may serve as age predictors due to their accurate correlation with time [31–34]. A recent study identified age-predicting, clock CpGs, also in mice [35]. Age-associated global demethylation might be the cause or effect of the global relaxation of chromatin structure at gene promoters in old T cells which we postulate is connected with the increase of noisy transcription in A_3_GE. In support of this hypothesis, we recently observed that the mutant HTT (mHTT) expression elevated epigenomic alterations in HD T cells; DNase I refractory promoters became susceptible to the nuclease in old HD T cells [7]. In order to demonstrate the generality of the relationship, i.e., the tripartite connection, between epigenetic drift, chromatin relaxation, and A_3_GE, it should be proven in other cell types through a more comprehensive and integrated panomics study involving the DNA methylome, DNase sequencing, targeted PCR sequencing, and the transcriptome.

T cells express both endogenous HTT and transgenic mHTT in HD mice [7]. HTT plays various roles in blood cell production and function [36]. A recent study showed that mHTT levels in patient peripheral T cells correlated with HD progression as well as brain atrophy rates [37] suggesting that mHTT expression and the enhanced A_3_GE found here in peripheral T cells have a potential relevance to the pathogenic and clinical events in the brain of HD patients. Similarly, we demonstrated that A_3_GE in T cells is aggravated in the HD genetic background, and this is associated with accelerated aging in these cells (Fig. 2C). In line with this, accelerated epigenetic aging was observed in the brain of HD patients [38,] and a significant reduction of telomere length was recently detected in the blood of HD patients [39]. In the latter study, other neurodegenerative disorders such as ataxia telangiectasia and dementia, in which accelerated telomere shortening has already been known [40–46], were also included for comparison with HD blood, but the reduction in the relative T/S ratio was shown to be the most significant in HD samples. Thus, although the precise mechanism for the telomere attrition in HD is unknown, the verification with larger number of HD samples in future study will ensure the use of relative T/S ratio as a biomarker for neuronal diseases. Our T/S ratio regression curve, Fig. 2B, indicates that the HD T cells at 16-19 months of age actually correspond to about 30-month old T cells. This accelerated aging intensified the A_3_GE in HD T cells, and the exacerbated A_3_GE could be a route for HD patients to pathophysiological transition or aggravation. We propose that perturbation of epigenome integrity [6,25] and the resulting increase of A_3_GE contributes mechanistically to neurological pathogenic transition in the mHTT genetic background.

As shown in Fig. 1, brain samples exhibited no evident A_3_GE. This was unexpected as brain tissue expressed the endogenous *Htt* gene strongly, two-fold higher than T cells (1.272 ± 0.173 vs. 0.589 ± 0.178), and we observed a similar difference in the expression level of human *mHTT* transgene between brain and T cells in YAC128 mice (data not shown). Furthermore, the brain sample analyzed in this study was the striatum, the primary site of damage in HD [47]. If the mHTT had directly caused A_3_GE, the striatum should have been the most conspicuous region for A_3_GE. Therefore, the finding that A_3_GE occurred in T cells only made no sense. A clue to an explanation was found in the wRMSD analysis. The T cell may be subject to an innately less restrictive gene regulation from a young age in that the wRMSD value was two-fold or more higher in the T cells compared with in the brain (0.0513 vs. 0.0229 in wRMSD; Fig. 1D). Furthermore, T cells undergo A_3_GE without mHTT expression (Fig. 1C). These observations suggest that the T cell has an innate plasticity and susceptibility to transcriptional regulation and alteration so that A_3_GE may be accelerated even with a relatively small amount of mHTT in the T cells.

The CD4+ T cell subset, a mixture of naïve and memory T cells, makes up > 40% of the splenic immune cell population [48]. Total T cell numbers in the spleen and the CD4/CD8 subset ratio are known to be least affected by aging [49]. The splenic CD4+ T cell population consists of naïve cells and memory cells at 2:8 ratio in humans [50] which changes with age [51]. The naïve T cells are depleted over a lifetime of encounters with acute and chronic pathogens, leading to a lifelong accumulation of memory cells that appears well preserved in aging (for review, see [52,53]), which raised the possibility that the A_3_GE in old T cells resulted from the change of the CD4+ T cell population ratio. It is difficult to draw a causal relationship between T cell A_3_GE and the cellular compositional change, and we assume that the effect would be minimal, given that the naïve cells occupy only one-fifth part of the splenic population in young human spleen [50] and, more importantly, the naïve CD4+ T cells show no evidence of decreasing with age in the mouse spleen, indicating that aging affects naïve T cell maintenance fundamentally differently in mice and humans [54].

Meanwhile, we should admit that our study has two weaknesses. One is the small sample size as we used only three or four mice per age group. The inclusion of more samples in the analysis would further corroborate the theory of age-related bimodal transcriptional drift we here proposed. The other is the use of single HD model moice only; other HD model mice stay unknown if they exhibit a similar accelerated aging and transcriptional drift as the YAC128 model. There are several HD model mice with different genetic constitution of *HTT/Htt* mutation in the mouse genome [55,56]. For instance, R6/2, which is one of the first HD mouse models, expresses N-terminal mHTT fragment and develops severe symptoms than other HD models [57]. Another HD model is the knock-in mice [58] which display a very late-onset phenotype in contrast to the R6/2 model [59]. Because of these phenotypic differences among the different HD model mice, it should be very careful of presuming the same extent of age-associated transcriptional change in these HD mice as in the YAC128 mice.

In conclusion, the failure to maintain cellular homeostasis with age might be expected to result in a global dysregulation of transcription. We asked the question of how well and consistently the quantitative transcriptome structure established in early life is maintained with age. We answered in this paper that transcriptional alterations become progressively larger as a function of age in T cells, and this trend results in a reduction in the differences of transcriptional activity between genes under expression.

## MATERIALS AND METHODS

### Ethics statement

This study was carried out in strict accordance with the recommendations in the Guide for the Care and Use of Laboratory Animals of the National Livestock Research Institute of Korea. The protocol was approved by the Committee on the Ethics of Animal Experiments of the Korea Research Institute of Bioscience and Biotechnology (KRIBB) and by Hanyang Institutional Animal Care and Use Committee (HY-IACUC-09-017).

### HD model mice and tissue preparation

YAC128 mice (FVB-Tg(YAC128)53Hay/J; Jackson Laboratory) which contains 128 CAG repeats of the human *HTT* gene [47] and wild-type littermates were kept in a 12 hour light-dark cycle with unrestricted access to food and water. Transgene in YAC128 was detected by PCR genotyping using primers 5’-CCGCTCAGGTTCTGCTTTTA-3’ and 5’-TGGAAGGACTTGAGGGACTC-3’. We used a total 7 of female wild-type littermate mice at 16-19 months (*n*=4) and 2 months (*n*=3) of age, and a total 7 of female YAC128 HD mice at 16-19 months (*n*=3) and 2 months (*n*=4).

The method for isolation of splenic CD4+ T cells and macrophages was described in detail elsewhere [7]. Brains were dissected to obtain striatal tissues and immediately after isolation, samples were frozen in dry ice and stored at -80°C deep-freezer. For skeletal muscles, tissues were obtained from hind legs and homogenized using Biomasher II (Biomasher) prior to total RNA extraction. cDNAs were generated from 1 μg of total RNA by incubating with Super-Script III (Invitrogen), oligo(dT)_20_ (Invitrogen), and random-hexamers (Invitrogen) at 50°C for 1 hour followed by enzyme inactivation at 75°C for 10 min.

### Multiplex PCR

Detection of nucleotide inter-species variation (ISVs) present in target genes between mouse mRNA and rat genomic DNA sequences through sequence alignment via BLAST, and primer design for the ISV-containing homologous sequence stretches are described in detail elsewhere [7]. In total, 285 primer pairs targeting 175 genes were split into 10 groups composed of 28~29 primer pairs and used in multiplexed PCR with 15 ng of mouse cDNA and 2 ng of rat gDNA as templates in following conditions; 95°C for 15 min, then at 95°C/20 sec, at 57°C /40 sec, at 72°C/1 min for 45 cycles and 72°C for 5 min. All experiments were duplicated for each sample.

### Amplicon library production and next generation sequencing

For construction of amplicon sequencing libraries for Illumina sequencing platform, PCR products of each sample generated by 10 groups of primer sets were mixed together and purified using Expin purification kit (GeneAll). The amplicon ends were phosphorylated with T4 polynucleotide kinase (NEB) by incubating at 37°C for 30 min, and were ligated with NGS adapters using T4 DNA ligase (Solgent) at 37°C for 2 hour. Finally, index PCR was conducted to complete Illumina sequencing libraries. The index PCR conditions are as follows; after 98°C for 15 min, proceeded to 20 cycles of 98°C/20 sec, 68°C/30 sec, and 72°C/1 min, followed by a final extension at 72°C for 5 min. The amplified libraries were purified and sequenced using HiSeq 2500. The barcoded sequencing libraries were pooled with equivalent amount and subjected to a multiple parallel sequence using Illumina HiSeq 2500 platform.

### wRMSD analysis

In order to quantify young-old difference in expression levels, we calculated the root mean square deviation (RMSD) of old samples from young samples. To minimize the bias that could result from a measurement error of gene expression profile of a group with a low coefficient of variation (CV, the deviation of each gene expression level from the mean) was weighted with the CV of the gene in the group [14–16]. The wRMSD was obtained with the equation,

$$wRMSD=\sqrt{\sum {w_{i}∙({Em}_{i}-E_{i})}^{2}}$$

where wi, Emi, and Ei are the values obtained from the ith gene of interest for the weight of the mean square deviation of the gene expression, the reference expression level (e.g., the mean expression level in the group), and the expression level of the gene, respectively. The weight (wi) is the proportion of the CV for the expression level of the ith gene to the sum of CV for those of all genes in the group and was obtained with the equation,

$$w_{i}=\frac{{CV}_{i}}{\sum {CV}_{i}}$$.

### Telomere length assay by qPCR

Genomic DNA was extracted from MACS-purified splenic T cells using DNeasy Blood & Tissue Kits (QIAGEN). For quantitative real-time PCR-mediated Telomere length assay [20], acidic ribosomal phosphoprotein P0 (*36B4*) single-copy gene was used as a standard. Primers used were 5′-CGGTTTGTTTGGGTTTGGGTTTGGGTTTGGGTTTGGGTT-3′ and 5′-GGCTTGCCTTACCCTTACCCTTACCCTTACCCTTACCCT-3’ for telomeric sequence, and 5'-ACTGGTCTAGGACCCGAGAAG-3' and 5'-TCAATGGTGCCTCTGGAGATT-3' for *36B4* gene sequence. Quantitative real-time PCR was performed using 10 ng of genomic DNA and 2X Power SYBR Green PCR Master Mix (ABI) in QuantStudio 3 Real-Time PCR System (ABI). Real-time PCR condition was set as 95°C /15 sec, 55°C /1 min and 72°C /1 min. Telomere to single copy gene (T/S) ratio is calculated as

$${{[2}^{Ct\left(telomeres\right)}/{\;2}^{Ct\left(36B4\right)}]}^{-1}=2^{-ÄCt}$$.

### Target gene expression estimation

Prior to the aligning of sequence reads, a custom reference genome was generated by gathering mouse mRNA sequences (mm10) of 260 target genes, and the reference was indexed by ‘bowtie2-build’ [60]. The raw sequencing reads were groomed to remove unwanted sequences and low quality bases using ‘Trim_galore (https://www.bioinformatics.babraham.ac.uk/projects/trim_galore/)’. The trimmed reads were mapped on the custom genome using ‘bowtie2’ with default parameters.

For quantification of mouse and rat read counts, sequence variation analyses were performed according to ‘The genome analysis toolkit (GATK)’ pipeline [61] with some modifications. The mapped reads were sorted by ‘Picard’ (https://broadinstitute.github.io/picard/), and sequence variations (SNVs) were called using ‘UnifiedGenotyper (GATK)’ with the down-sampling option disabled. Due to the nature of amplicon sequencing, duplicated reads were preserved throughout following analyses. Since INDEL was not included for targets, the INDEL realignment step was skipped. The resulting VCF files were filtered to obtain read count information only at pre-defined the SNVs sites. The number of reference (mouse) and alternative (rat) sequences was counted at the filtered SNV sites using home-brew bash scripts. The count data were corrected for deviations in the fractional quantities as previously described [8]. Finally, gene expression levels were estimated by calculating relative read counts of mouse to rat (M/R ratio). To draw PCA plots, raw count data for all samples were combined and normalized by ‘DESeq’ [62]. Using ‘plotPCA’ function, the plots were generated.

### RNA-seq and data analysis

Total RNAs were extracted from T and blood cells of each age group using RNeasy Plus Mini Kit (Qiagen). Dynabeads mRNA DIRECT (Thermo) was used to isolate poly-A tailed RNAs from 2 μg of total RNAs. Each step was conducted according to the manufacturer's instruction. Isolated poly-A tailed RNAs were used to construct an RNAseq library using NEBNext Ultra RNA library prep kit for illumina (NEB). Isolated poly-A tailed RNAs were incubated at 94°C for 15 min for fragmentation. First strand cDNA was synthesized with fragmented RNAs and ProtoScript II Reverse Transcriptase. Second strand cDNA was synthesized with Second Strand Synthesis Enzyme Mix in the kit and purified. After end repair of the synthesized double-stranded DNAs using NEBNext End Prep Enzyme Mix in the condition of 20°C for 30 min and 65°C for 30 min, NEBNext Adaptor was added into the 3’ end adenylated DNA with Blunt/TA Ligase Master Mix and the mixture was incubated at 20°C for 15 min. DNA samples were enriched by 15 cycles of PCR reaction consisting of the purified ligates, NEBNext Q5 Hot Start HiFi PCR Master Mix 2X, and index primer. Each purification was conducted using Ampure XP beads (Beckman). Enriched DNA fragments were purified and sequenced using NextSeq 500.

Raw sequencing reads were trimmed to remove Illumina adapter sequences and low quality bases using ‘Trim_galore (https://www.bioinformatics.babraham.ac.uk/projects/trim_galore/)’, and the trimmed reads were aligned on the reference transcriptome (mm10) using STAR: ultrafast universal RNA-seq aligner [63]. For estimation of gene expression levels, we followed ‘cufflinks pipeline’ [64]. Transcriptome was assembled by ‘cufflinks’ and ‘cuffmerge’, and gene expression levels were estimated and by ‘cuffquant’, and finally, a matrix of gene expression levels in RPKM was generated by ‘cuffnorm’.

## Supplementary Material

Supplementary Figures

Supplementary Data File 1

## References

[1] Brunet A, Berger SL. Epigenetics of aging and aging-related disease. J Gerontol A Biol Sci Med Sci. 2014 (Suppl 1); 69:S17–20. 10.1093/gerona/glu042 24833581 PMC4022130

[2] Feser J, Tyler J. Chromatin structure as a mediator of aging. FEBS Lett. 2011; 585:2041–48. 10.1016/j.febslet.2010.11.016 21081125 PMC3988783

[3] Kennedy BK, Berger SL, Brunet A, Campisi J, Cuervo AM, Epel ES, Franceschi C, Lithgow GJ, Morimoto RI, Pessin JE, Rando TA, Richardson A, Schadt EE, et al. Geroscience: linking aging to chronic disease. Cell. 2014; 159:709–13. 10.1016/j.cell.2014.10.039 25417146 PMC4852871

[4] Moskalev AA, Aliper AM, Smit-McBride Z, Buzdin A, Zhavoronkov A. Genetics and epigenetics of aging and longevity. Cell Cycle. 2014; 13:1063–77. 10.4161/cc.28433 24603410 PMC4013158

[5] López-Otín C, Blasco MA, Partridge L, Serrano M, Kroemer G. The hallmarks of aging. Cell. 2013; 153:1194–217. 10.1016/j.cell.2013.05.039 23746838 PMC3836174

[6] Stegeman R, Weake VM. Transcriptional Signatures of Aging. J Mol Biol. 2017; 429:2427–37. 10.1016/j.jmb.2017.06.019 28684248 PMC5662117

[7] Park M, Min B, Jeon K, Cho S, Park JS, Kim J, Jeon J, Song J, Kim S, Jeong S, Seo H, Kang YK. Age-associated chromatin relaxation is enhanced in Huntington’s disease mice. Aging (Albany NY). 2017; 9:803–22. 10.18632/aging.101193 28288000 PMC5391233

[8] Oh SA, Yang I, Hahn Y, Kang YK, Chung SK, Jeong S. SiNG-PCRseq: accurate inter-sequence quantification achieved by spiking-in a neighbor genome for competitive PCR amplicon sequencing. Sci Rep. 2015; 5:11879. 10.1038/srep11879 26144254 PMC4491706

[9] Moffitt H, McPhail GD, Woodman B, Hobbs C, Bates GP. Formation of polyglutamine inclusions in a wide range of non-CNS tissues in the HdhQ150 knock-in mouse model of Huntington’s disease. PLoS One. 2009; 4:e8025. 10.1371/journal.pone.0008025 19956633 PMC2778556

[10] van der Burg JM, Björkqvist M, Brundin P. Beyond the brain: widespread pathology in Huntington’s disease. Lancet Neurol. 2009; 8:765–74. 10.1016/S1474-4422(09)70178-4 19608102

[11] Björkqvist M, Wild EJ, Thiele J, Silvestroni A, Andre R, Lahiri N, Raibon E, Lee RV, Benn CL, Soulet D, Magnusson A, Woodman B, Landles C, et al. A novel pathogenic pathway of immune activation detectable before clinical onset in Huntington’s disease. J Exp Med. 2008; 205:1869–77. 10.1084/jem.20080178 18625748 PMC2525598

[12] Diamanti D, Lahiri N, Tarditi A, Magnoni L, Fondelli C, Morena E, Malusa F, Pollio G, Diodato E, Tripepi G, Tabrizi SJ, Caricasole A, Mori E. Reference genes selection for transcriptional profiling in blood of HD patients and R6/2 mice. J Huntingtons Dis. 2013; 2:185–200. 10.3233/JHD-120042 25063515

[13] Borovecki F, Lovrecic L, Zhou J, Jeong H, Then F, Rosas HD, Hersch SM, Hogarth P, Bouzou B, Jensen RV, Krainc D. Genome-wide expression profiling of human blood reveals biomarkers for Huntington’s disease. Proc Natl Acad Sci USA. 2005; 102:11023–28. 10.1073/pnas.0504921102 16043692 PMC1182457

[14] Kwon S, Park JS, Kang YK. Differences in the gene expression profiles of slow- and fast-forming pre-induced pluripotent stem cell colonies. Stem Cell Int.. 2015; 2015:195928. 10.1155/2015/195928 25945097 PMC4405011

[15] Kwon S, Jeong S, Park JS, Kang YK. Quantifying difference in gene expression profile between bovine blastocysts derived by in vitro fertilization and somatic cell nuclear transfer. Gene Expr Patterns. 2015; 19:14–20. 10.1016/j.gep.2015.05.005 26101995

[16] Kwon S, Jeong S, Jeong YS, Park JS, Cui XS, Kim NH, Kang YK. Assessment of difference in gene expression profile between embryos of different derivations. Cell Reprogram. 2015; 17:49–58. 10.1089/cell.2014.0057 25549061 PMC4312879

[17] Valdes AM, Andrew T, Gardner JP, Kimura M, Oelsner E, Cherkas LF, Aviv A, Spector TD. Obesity, cigarette smoking, and telomere length in women. Lancet. 2005; 366:662–64. 10.1016/S0140-6736(05)66630-5 16112303

[18] Brouilette S, Singh RK, Thompson JR, Goodall AH, Samani NJ. White cell telomere length and risk of premature myocardial infarction. Arterioscler Thromb Vasc Biol. 2003; 23:842–46. 10.1161/01.ATV.0000067426.96344.32 12649083

[19] Cawthon RM. Telomere length measurement by a novel monochrome multiplex quantitative PCR method. Nucleic Acids Res. 2009; 37:e21. 10.1093/nar/gkn1027 19129229 PMC2647324

[20] Cawthon RM. Telomere measurement by quantitative PCR. Nucleic Acids Res. 2002; 30:e47. 10.1093/nar/30.10.e47 12000852 PMC115301

[21] Harries LW, Hernandez D, Henley W, Wood AR, Holly AC, Bradley-Smith RM, Yaghootkar H, Dutta A, Murray A, Frayling TM, Guralnik JM, Bandinelli S, Singleton A, et al. Human aging is characterized by focused changes in gene expression and deregulation of alternative splicing. Aging Cell. 2011; 10:868–78. 10.1111/j.1474-9726.2011.00726.x 21668623 PMC3173580

[22] Mastrokolias A, Ariyurek Y, Goeman JJ, van Duijn E, Roos RA, van der Mast RC, van Ommen GB, den Dunnen JT, ’t Hoen PA, van Roon-Mom WM. Huntington’s disease biomarker progression profile identified by transcriptome sequencing in peripheral blood. Eur J Hum Genet. 2015; 23:1349–56. 10.1038/ejhg.2014.281 25626709 PMC4592077

[23] Hu Y, Chopra V, Chopra R, Locascio JJ, Liao Z, Ding H, Zheng B, Matson WR, Ferrante RJ, Rosas HD, Hersch SM, Scherzer CR. Transcriptional modulator H2A histone family, member Y (H2AFY) marks Huntington disease activity in man and mouse. Proc Natl Acad Sci USA. 2011; 108:17141–46. 10.1073/pnas.1104409108 21969577 PMC3193232

[24] Runne H, Kuhn A, Wild EJ, Pratyaksha W, Kristiansen M, Isaacs JD, Régulier E, Delorenzi M, Tabrizi SJ, Luthi-Carter R. Analysis of potential transcriptomic biomarkers for Huntington’s disease in peripheral blood. Proc Natl Acad Sci USA. 2007; 104:14424–29. 10.1073/pnas.0703652104 17724341 PMC1964868

[25] Booth LN, Brunet A. The Aging Epigenome. Mol Cell. 2016; 62:728–44. 10.1016/j.molcel.2016.05.013 27259204 PMC4917370

[26] O’Sullivan RJ, Kubicek S, Schreiber SL, Karlseder J. Reduced histone biosynthesis and chromatin changes arising from a damage signal at telomeres. Nat Struct Mol Biol. 2010; 17:1218–25. 10.1038/nsmb.1897 20890289 PMC2951278

[27] Jeyapalan JC, Ferreira M, Sedivy JM, Herbig U. Accumulation of senescent cells in mitotic tissue of aging primates. Mech Ageing Dev. 2007; 128:36–44. 10.1016/j.mad.2006.11.008 17116315 PMC3654105

[28] Bahar R, Hartmann CH, Rodriguez KA, Denny AD, Busuttil RA, Dollé ME, Calder RB, Chisholm GB, Pollock BH, Klein CA, Vijg J. Increased cell-to-cell variation in gene expression in ageing mouse heart. Nature. 2006; 441:1011–14. 10.1038/nature04844 16791200

[29] Jung M, Pfeifer GP. Aging and DNA methylation. BMC Biol. 2015; 13:7. 10.1186/s12915-015-0118-4 25637097 PMC4311512

[30] Teschendorff AE, West J, Beck S. Age-associated epigenetic drift: implications, and a case of epigenetic thrift? Hum Mol Genet. 2013; 22:R7–15. 10.1093/hmg/ddt375 23918660 PMC3782071

[31] Bocklandt S, Lin W, Sehl ME, Sánchez FJ, Sinsheimer JS, Horvath S, Vilain E. Epigenetic predictor of age. PLoS One. 2011; 6:e14821. 10.1371/journal.pone.0014821 21731603 PMC3120753

[32] Koch CM, Wagner W. Epigenetic-aging-signature to determine age in different tissues. Aging (Albany NY). 2011; 3:1018–27. 10.18632/aging.100395 22067257 PMC3229965

[33] Hannum G, Guinney J, Zhao L, Zhang L, Hughes G, Sadda S, Klotzle B, Bibikova M, Fan JB, Gao Y, Deconde R, Chen M, Rajapakse I, et al. Genome-wide methylation profiles reveal quantitative views of human aging rates. Mol Cell. 2013; 49:359–67. 10.1016/j.molcel.2012.10.016 23177740 PMC3780611

[34] Horvath S. DNA methylation age of human tissues and cell types. Genome Biol. 2013; 14:R115. 10.1186/gb-2013-14-10-r115 24138928 PMC4015143

[35] Stubbs TM, Bonder MJ, Stark AK, Krueger F, von Meyenn F, Stegle O, Reik W, Reik W, and BI Ageing Clock Team. Multi-tissue DNA methylation age predictor in mouse. Genome Biol. 2017; 18:68. 10.1186/s13059-017-1203-5 28399939 PMC5389178

[36] Metzler M, Helgason CD, Dragatsis I, Zhang T, Gan L, Pineault N, Zeitlin SO, Humphries RK, Hayden MR. Huntingtin is required for normal hematopoiesis. Hum Mol Genet. 2000; 9:387–94. 10.1093/hmg/9.3.387 10655548

[37] Weiss A, Träger U, Wild EJ, Grueninger S, Farmer R, Landles C, Scahill RI, Lahiri N, Haider S, Macdonald D, Frost C, Bates GP, Bilbe G, et al. Mutant huntingtin fragmentation in immune cells tracks Huntington’s disease progression. J Clin Invest. 2012; 122:3731–36. 10.1172/JCI64565 22996692 PMC3461928

[38] Horvath S, Langfelder P, Kwak S, Aaronson J, Rosinski J, Vogt TF, Eszes M, Faull RL, Curtis MA, Waldvogel HJ, Choi OW, Tung S, Vinters HV, et al. Huntington’s disease accelerates epigenetic aging of human brain and disrupts DNA methylation levels. Aging (Albany NY). 2016; 8:1485–512. 10.18632/aging.101005 27479945 PMC4993344

[39] Kota LN, Bharath S, Purushottam M, Moily NS, Sivakumar PT, Varghese M, Pal PK, Jain S. Reduced telomere length in neurodegenerative disorders may suggest shared biology. J Neuropsychiatry Clin Neurosci. 2015; 27:e92–96. 10.1176/appi.neuropsych.13100240 25541866

[40] Grodstein F, van Oijen M, Irizarry MC, Rosas HD, Hyman BT, Growdon JH, De Vivo I. Shorter telomeres may mark early risk of dementia: preliminary analysis of 62 participants from the nurses’ health study. PLoS One. 2008; 3:e1590. 10.1371/journal.pone.0001590 18795148 PMC2536511

[41] Hochstrasser T, Marksteiner J, Humpel C. Telomere length is age-dependent and reduced in monocytes of Alzheimer patients. Exp Gerontol. 2012; 47:160–63. 10.1016/j.exger.2011.11.012 22178633 PMC3278593

[42] Honig LS, Kang MS, Schupf N, Lee JH, Mayeux R. Association of shorter leukocyte telomere repeat length with dementia and mortality. Arch Neurol. 2012; 69:1332–39. 10.1001/archneurol.2012.1541 22825311 PMC3622729

[43] Thomas P, O’ Callaghan NJ, Fenech M. Telomere length in white blood cells, buccal cells and brain tissue and its variation with ageing and Alzheimer’s disease. Mech Ageing Dev. 2008; 129:183–90. 10.1016/j.mad.2007.12.004 18242664

[44] von Zglinicki T, Serra V, Lorenz M, Saretzki G, Lenzen-Grossimlighaus R, Gessner R, Risch A, Steinhagen-Thiessen E. Short telomeres in patients with vascular dementia: an indicator of low antioxidative capacity and a possible risk factor? Lab Invest. 2000; 80:1739–47. 10.1038/labinvest.3780184 11092534

[45] Metcalfe JA, Parkhill J, Campbell L, Stacey M, Biggs P, Byrd PJ, Taylor AM. Accelerated telomere shortening in ataxia telangiectasia. Nat Genet. 1996; 13:350–53. 10.1038/ng0796-350 8673136

[46] Tchirkov A, Lansdorp PM. Role of oxidative stress in telomere shortening in cultured fibroblasts from normal individuals and patients with ataxia-telangiectasia. Hum Mol Genet. 2003; 12:227–32. 10.1093/hmg/ddg023 12554677

[47] Slow EJ, van Raamsdonk J, Rogers D, Coleman SH, Graham RK, Deng Y, Oh R, Bissada N, Hossain SM, Yang YZ, Li XJ, Simpson EM, Gutekunst CA, et al. Selective striatal neuronal loss in a YAC128 mouse model of Huntington disease. Hum Mol Genet. 2003; 12:1555–67. 10.1093/hmg/ddg169 12812983

[48] Noubade R, Wong K, Ota N, Rutz S, Eidenschenk C, Valdez PA, Ding J, Peng I, Sebrell A, Caplazi P, DeVoss J, Soriano RH, Sai T, et al. NRROS negatively regulates reactive oxygen species during host defence and autoimmunity. Nature. 2014; 509:235–39. 10.1038/nature13152 24739962

[49] Linton PJ, Dorshkind K. Age-related changes in lymphocyte development and function. Nat Immunol. 2004; 5:133–39. 10.1038/ni1033 14749784

[50] Langeveld M, Gamadia LE, ten Berge IJ. T-lymphocyte subset distribution in human spleen. Eur J Clin Invest. 2006; 36:250–56. 10.1111/j.1365-2362.2006.01626.x 16620287

[51] Haynes L, Swain SL, Cambier J, Fulder R. Aging and immune function. Summary of a workshop held at Trudeau Institute, Saranac Lake, NY. Mech Ageing Dev. 2005; 126:822–25. 10.1016/j.mad.2005.02.007 15981319

[52] Nikolich-Žugich J. Aging of the T cell compartment in mice and humans: from no naive expectations to foggy memories. J Immunol. 2014; 193:2622–29. 10.4049/jimmunol.1401174 25193936 PMC4157314

[53] Nikolich-Zugich J. T cell aging: naive but not young. J Exp Med. 2005; 201:837–40. 10.1084/jem.20050341 15781575 PMC2213096

[54] den Braber I, Mugwagwa T, Vrisekoop N, Westera L, Mögling R, de Boer AB, Willems N, Schrijver EH, Spierenburg G, Gaiser K, Mul E, Otto SA, Ruiter AF, et al. Maintenance of peripheral naive T cells is sustained by thymus output in mice but not humans. Immunity. 2012; 36:288–97. 10.1016/j.immuni.2012.02.006 22365666

[55] Ehrnhoefer DE, Butland SL, Pouladi MA, Hayden MR. Mouse models of Huntington disease: variations on a theme. Dis Model Mech. 2009; 2:123–29. 10.1242/dmm.002451 19259385 PMC2650190

[56] Menalled LB, Chesselet MF. Mouse models of Huntington’s disease. Trends Pharmacol Sci. 2002; 23:32–39. 10.1016/S0165-6147(00)01884-8 11804649

[57] Mangiarini L, Sathasivam K, Seller M, Cozens B, Harper A, Hetherington C, Lawton M, Trottier Y, Lehrach H, Davies SW, Bates GP. Exon 1 of the HD gene with an expanded CAG repeat is sufficient to cause a progressive neurological phenotype in transgenic mice. Cell. 1996; 87:493–506. 10.1016/S0092-8674(00)81369-0 8898202

[58] Shelbourne PF, Killeen N, Hevner RF, Johnston HM, Tecott L, Lewandoski M, Ennis M, Ramirez L, Li Z, Iannicola C, Littman DR, Myers RM. A Huntington’s disease CAG expansion at the murine Hdh locus is unstable and associated with behavioural abnormalities in mice. Hum Mol Genet. 1999; 8:763–74. 10.1093/hmg/8.5.763 10196365

[59] Heng MY, Tallaksen-Greene SJ, Detloff PJ, Albin RL. Longitudinal evaluation of the Hdh(CAG)150 knock-in murine model of Huntington’s disease. J Neurosci. 2007; 27:8989–98. 10.1523/JNEUROSCI.1830-07.2007 17715336 PMC6672210

[60] Langdon WB. Performance of genetic programming optimised Bowtie2 on genome comparison and analytic testing (GCAT) benchmarks. BioData Min. 2015; 8:1. 10.1186/s13040-014-0034-0 25621011 PMC4304608

[61] McKenna A, Hanna M, Banks E, Sivachenko A, Cibulskis K, Kernytsky A, Garimella K, Altshuler D, Gabriel S, Daly M, DePristo MA. The Genome Analysis Toolkit: a MapReduce framework for analyzing next-generation DNA sequencing data. Genome Res. 2010; 20:1297–303. 10.1101/gr.107524.110 20644199 PMC2928508

[62] Anders S, Huber W. Differential expression analysis for sequence count data. Genome Biol. 2010; 11:R106. 10.1186/gb-2010-11-10-r106 20979621 PMC3218662

[63] Dobin A, Davis CA, Schlesinger F, Drenkow J, Zaleski C, Jha S, Batut P, Chaisson M, Gingeras TR. STAR: ultrafast universal RNA-seq aligner. Bioinformatics. 2013; 29:15–21. 10.1093/bioinformatics/bts635 23104886 PMC3530905

[64] Trapnell C, Roberts A, Goff L, Pertea G, Kim D, Kelley DR, Pimentel H, Salzberg SL, Rinn JL, Pachter L. Differential gene and transcript expression analysis of RNA-seq experiments with TopHat and Cufflinks. Nat Protoc. 2012; 7:562–78. 10.1038/nprot.2012.016 22383036 PMC3334321

