# Supplementary material for: Age-associated bimodal transcriptional drift reduces intergenic disparities in transcription
Source: Aging (Albany NY). 2018 Apr 27;10(4):789–807. doi: 10.18632/aging.101428 (PMC5940109; doi:10.18632/aging.101428)
Supplement: Supplementary Figures [file aging-10-101428-s001.pdf]

# SUPPLEMENTARY MATERIAL

Please browse the Full Text version to see **Supplementary Data File.** An excel file containing mapping rates and gene expression tables for all tissue types.

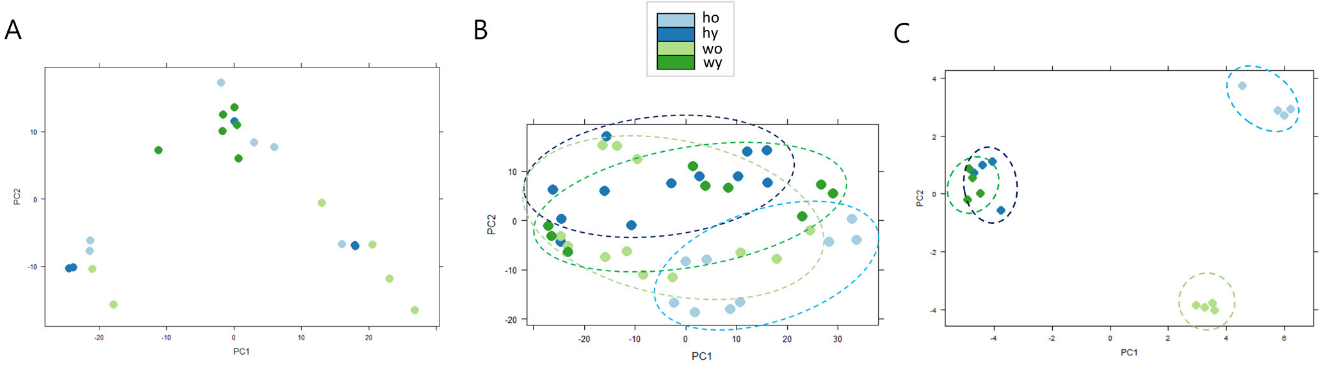


**Supplementary Figure S1.** Relationships between the epi-driver gene expression of young and old samples of muscle, brain, and T cells. Results of principal component analysis (PCA) of skeletal muscle (**A**), brain (striatum; (**B**), and splenic CD4+ T cells (**C**). Samples of different ages and genotypes are indicated by different colors: dark and light greens for young (wy) and old wild-type (wo) mice samples, and dark and light blues for young (hy) and old Huntington's disease (ho) mouse samples. Samples from the same tissues are circled by the indicated colored lines in **B** and **C**.

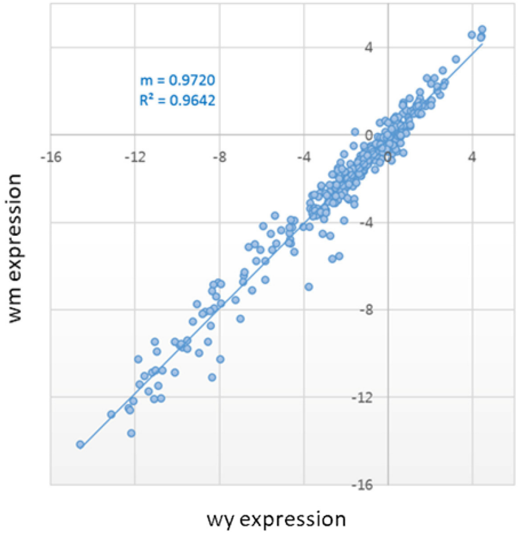


**Supplementary Figure S2.** Scatter plot of the epi-driver gene expression levels between young and 7-8 month old (wm) T cells of wild-type mice. The slope (m) of the regression curve (blue line) and the correlation value (R2, coefficient of determination) are shown.

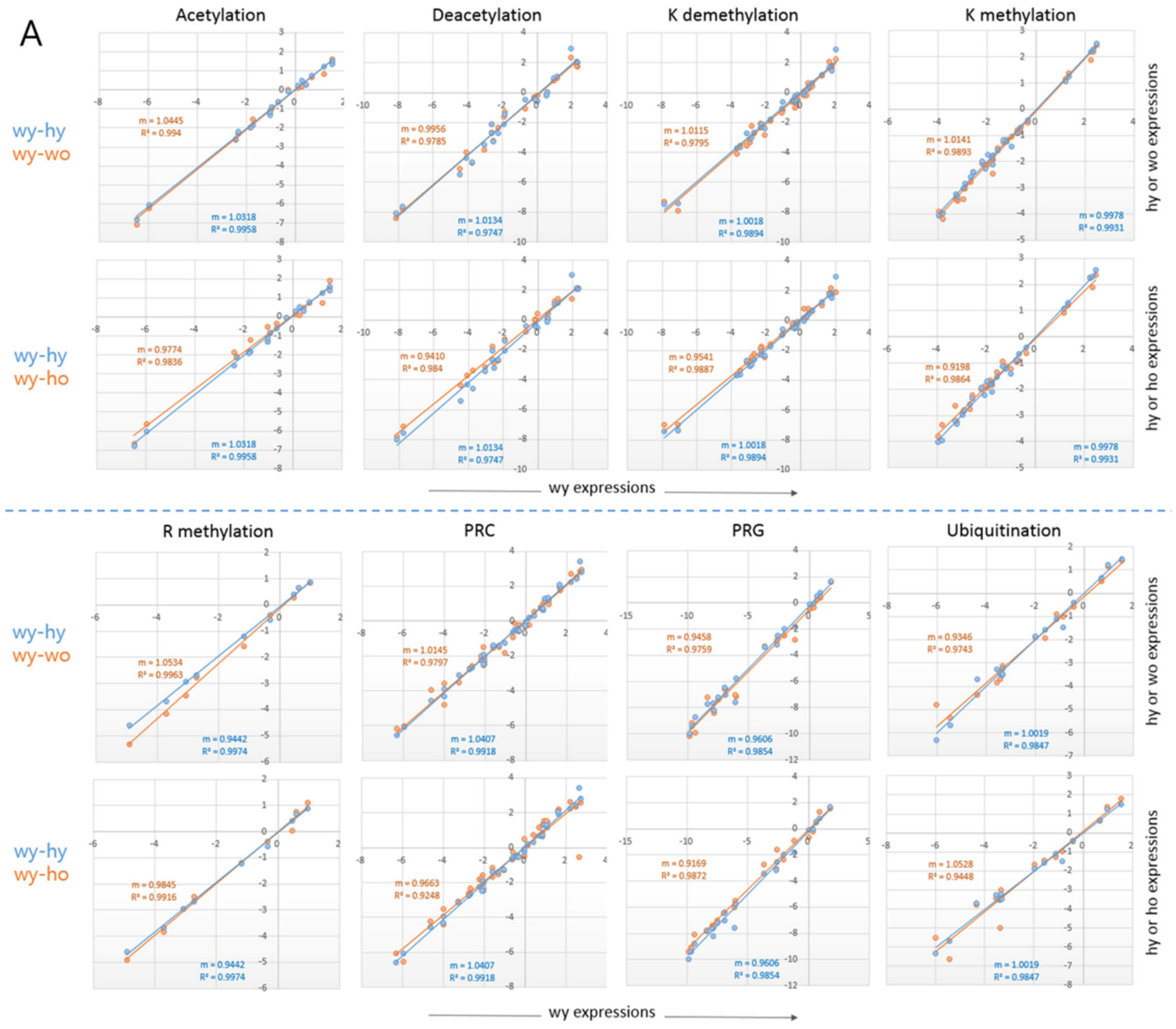


**Supplementary Figure S3A.** Comparisons of the expression levels of epi-driver genes between wy vs. hy (blue) and between wy vs. wo (orange); between wy vs. hy (blue) and wy vs. ho (orange) in muscle tissue (**A**) and brain tissue (**B**). Each scatter plot shows the slope (m) of the regression curve (blue and orange linear lines) and the correlation value (R2, coefficient of determination). PRC, Polycomb-repressive complex; PRG, PRC-regulated genes.

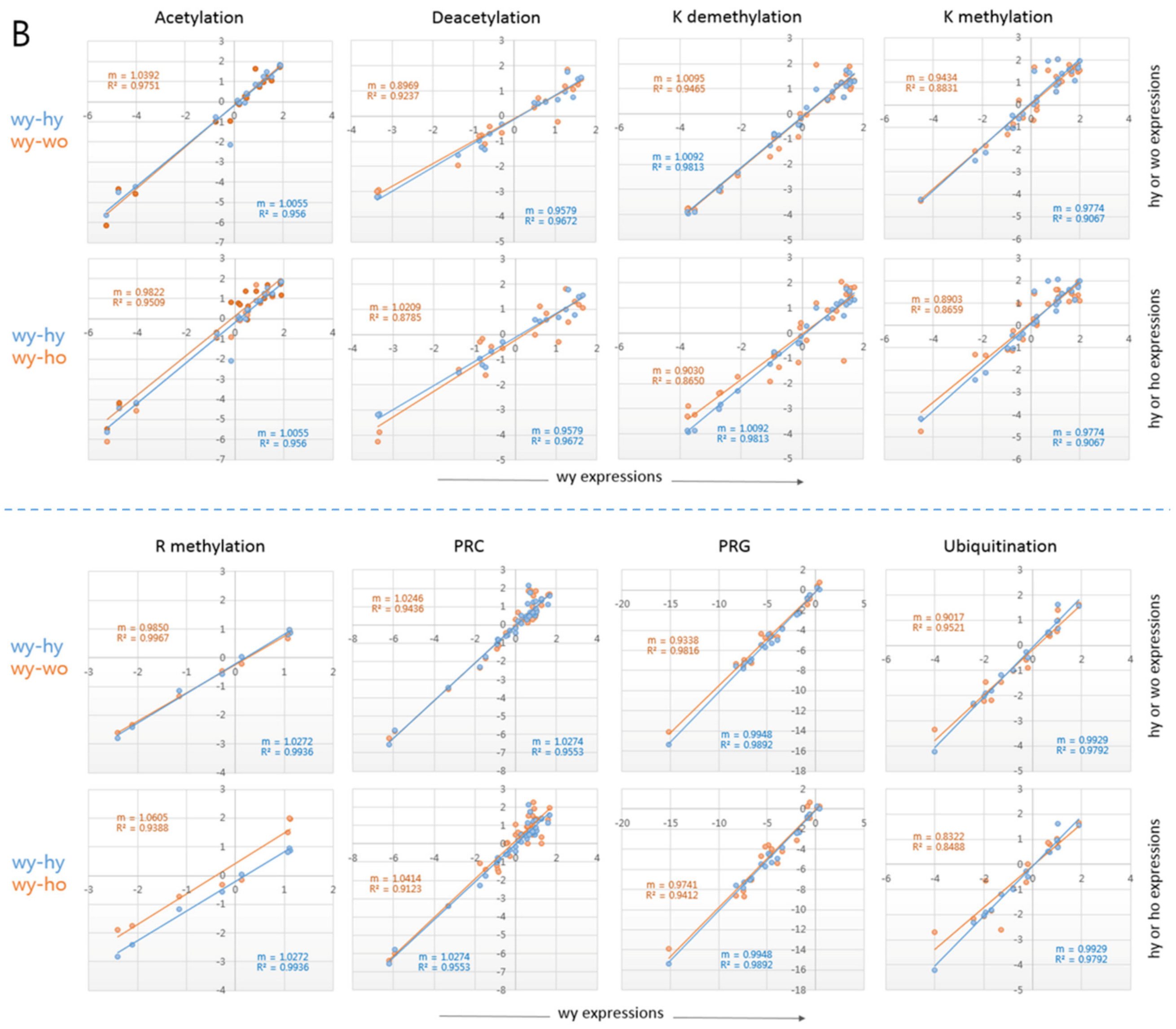


**Supplementary Figure S3B.** Comparisons of the expression levels of epi-driver genes between wy vs. hy (blue) and between wy vs. wo (orange); between wy vs. hy (blue) and wy vs. ho (orange) in muscle tissue (**A**) and brain tissue (**B**). Each scatter plot shows the slope (m) of the regression curve (blue and orange linear lines) and the correlation value (R2, coefficient of determination). PRC, Polycomb-repressive complex; PRG, PRC-regulated genes.

| Category[a] | | Slope | | | A3GE | | |
|---|---|---|---|---|---|---|---|
| | | wy-hy | wy-wo | wy-ho | wy-hy | wy-wo | wy-ho |
| Mu | Average | 0.999 | 1.0022 | 0.9641 | 0.02338 | 0.03319 | 0.0491 |
| | Acetylation | 1.0318 | 1.0445 | 0.9774 | 0.0318 | 0.0445 | 0.0226 |
| | Deacetylation | 1.0134 | 0.9956 | 0.941 | 0.0134 | 0.0044 | 0.059 |
| | K demethylation | 1.0018 | 1.015 | 0.9541 | 0.0018 | 0.015 | 0.0459 |
| | K methylation | 0.9978 | 1.0141 | 0.9198 | 0.0022 | 0.0141 | 0.0802 |
| | R methylation | 0.9442 | 1.0534 | 0.9845 | 0.0558 | 0.0534 | 0.0155 |
| | PRC | 1.0407 | 1.0145 | 0.9663 | 0.0407 | 0.0145 | 0.0337 |
| | PRG | 0.9606 | 0.9458 | 0.9169 | 0.0394 | 0.0542 | 0.0831 |
| | Ubiquitination | 1.0019 | 0.9346 | 1.0528 | 0.0019 | 0.0654 | 0.0528 |
| Br | Average | 0.999 | 0.966 | 0.9631 | 0.01829 | 0.05078 | 0.06763 |
| | Acetylation | 1.0055 | 1.0329 | 0.9822 | 0.0055 | 0.0329 | 0.0178 |
| | Deacetylation | 0.9579 | 0.8969 | 1.0209 | 0.0421 | 0.1031 | 0.0209 |
| | K demethylation | 1.0092 | 1.0095 | 0.903 | 0.0092 | 0.0095 | 0.097 |
| | K methylation | 0.9774 | 0.9434 | 0.8903 | 0.0226 | 0.0566 | 0.1097 |
| | R methylation | 1.0272 | 0.985 | 1.0605 | 0.0272 | 0.015 | 0.0605 |
| | PRC | 1.0274 | 1.0246 | 1.0414 | 0.0274 | 0.0246 | 0.0414 |
| | PRG | 0.9948 | 0.9338 | 0.9741 | 0.0052 | 0.0662 | 0.0259 |
| | Ubiquitination | 0.9929 | 0.9017 | 0.8322 | 0.0071 | 0.0983 | 0.1678 |
| Tc | Average | 1.0429 | 0.7066 | 0.2182 | 0.10189 | 0.30011 | 0.7818 |
| | Acetylation | 1.0502 | 0.5393 | 0.6815 | 0.0502 | 0.4607 | 0.3185 |
| | Deacetylation | 0.949 | 0.4861 | 0.2167 | 0.051 | 0.5139 | 0.7833 |
| | K demethylation | 0.9332 | 0.7556 | 0.1538 | 0.0668 | 0.2444 | 0.8462 |
| | K methylation | 0.9765 | 0.7232 | 0.2931 | 0.0235 | 0.2768 | 0.7069 |
| | R methylation | 1.0718 | 0.8462 | 0.1109 | 0.0718 | 0.1538 | 0.8891 |
| | PRC | 0.9052 | 0.6689 | 0.2281 | 0.0948 | 0.3311 | 0.7719 |
| | PRG | 1.2327 | 1.027 | 0.0209 | 0.2327 | 0.027 | 0.9791 |
| | Ubiquitination | 1.2243 | 0.6068 | 0.0406 | 0.2243 | 0.3932 | 0.9594 |

[a] Mu, muscle; Br, brain; Tc, T cell.

**Supplementary Figure S4.** Summary of slopes and A3GE in the scatter plots of epi-driver gene expression levels of different categories in the skeletal muscle (Mu), brain (Br), and T cells (Tc).

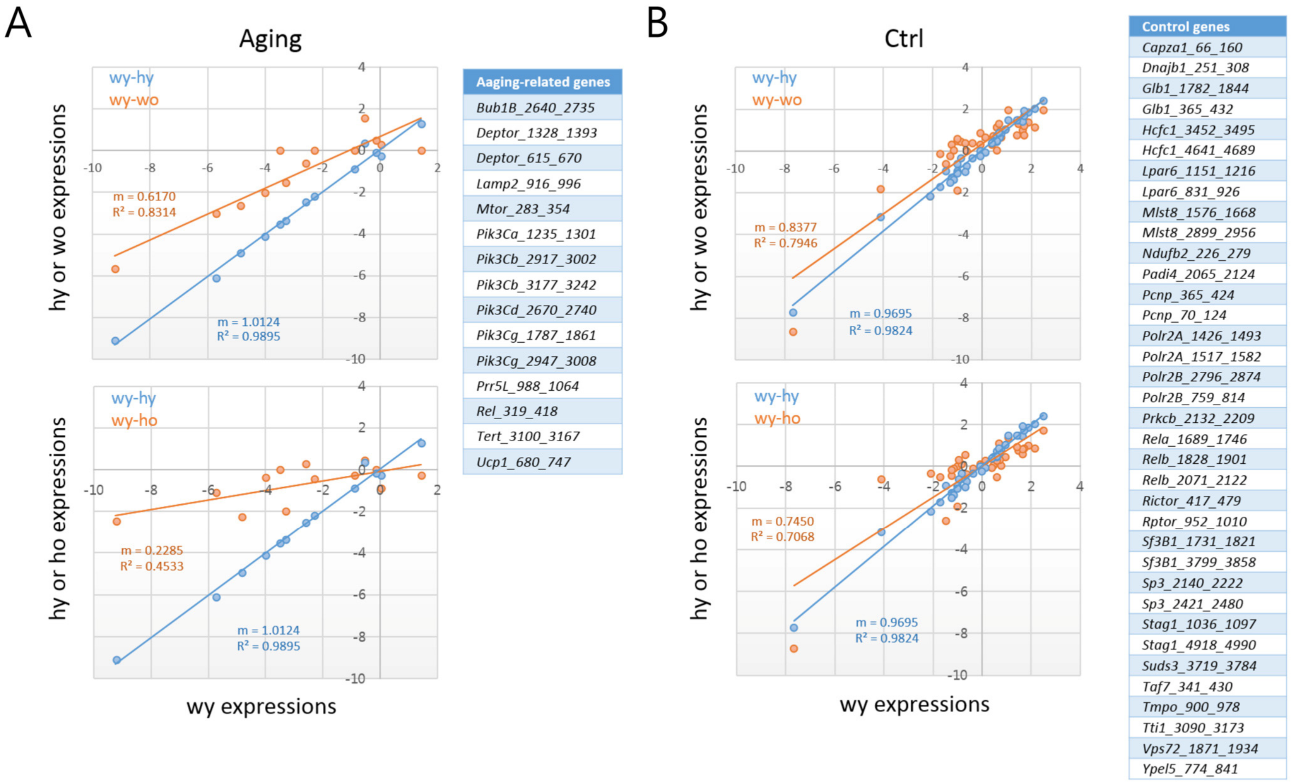


**Supplementary Figure S5.** Scatter plots comparing the expression levels of aging-related genes (**A**) and of a group of genes selected for relatively stable expression across different cell types (**B**) between the wy vs. hy (blue) and wy vs. wo (orange) or between wy vs. hy and wy vs. ho (orange) in T cells. Each plot shows the slope (m) of the regression curve (linear blue and orange lines) and the correlation value (R2, coefficient of determination).

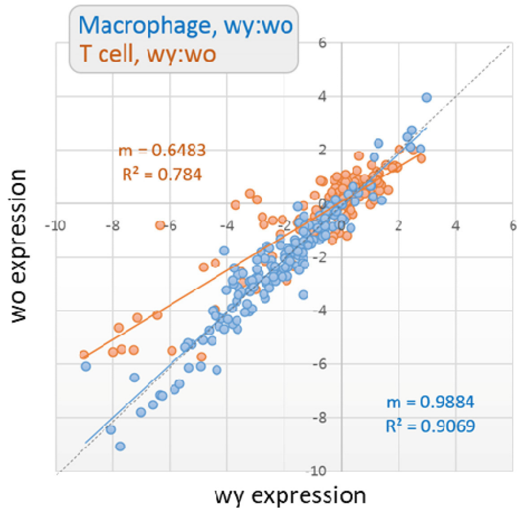


**Supplementary Figure S6.** Comparisons of expression levels of the epi-driver genes between young wild-type (wy) and old wild-type (wo) samples of splenic T cells (orange) and splenic macrophages (blue). Each plot shows the slope (m) of the regression curve (linear blue and orange lines) and the correlation value (R2, coefficient of determination).

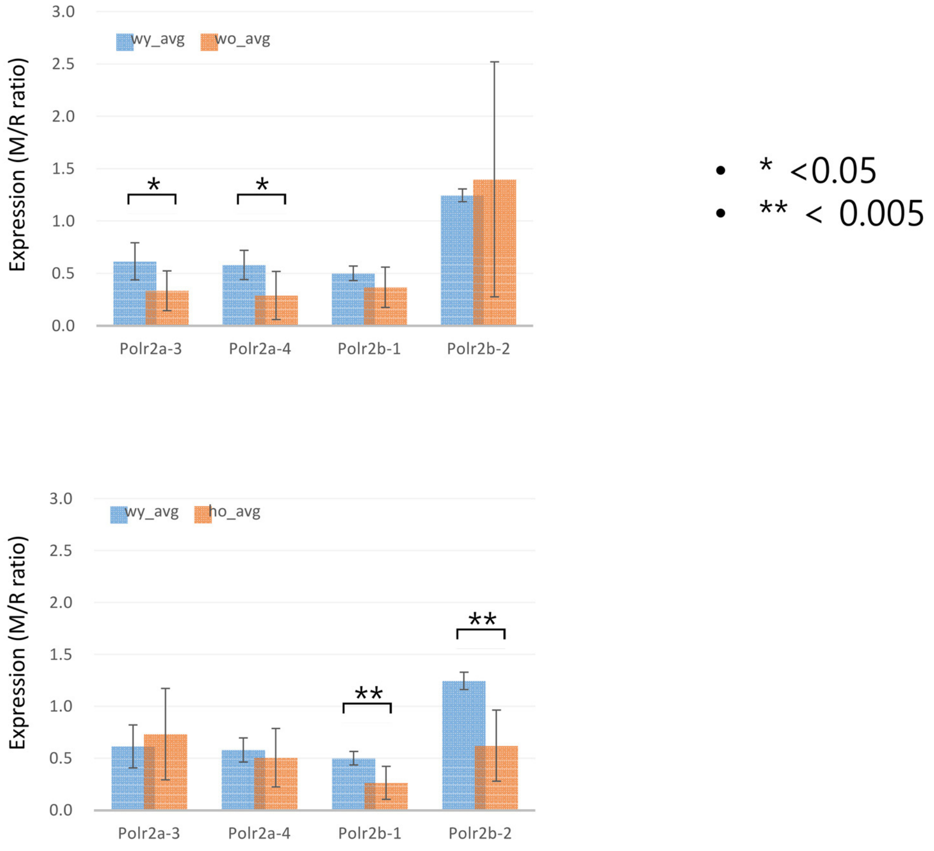


**Supplementary Figure S7.** Expression levels of RNA polymerase II subunit A (Polr2a) and B (Polr2b) genes in young and old wild-type (wy and wo) T cells and young and old HD (hy and ho) T cells. Expression level was measured by calculating the M/R ratio, the ratio of mouse read counts to rat read counts. Single and double asterisks denote a statistical significance at $p < 0.05$ and 0.005 (paired sample t-test), respectively. Error bars, standard deviations.

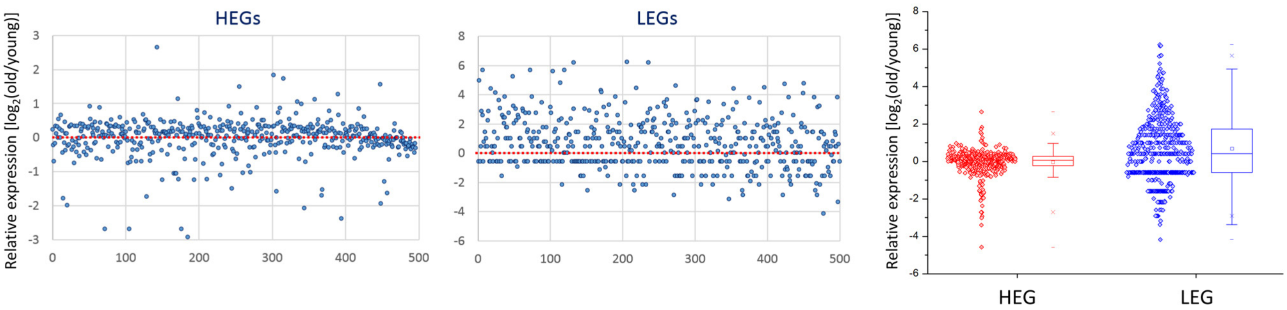


**Supplementary Figure S8.** A3GE in the transcriptomes of peripheral blood mononuclear cells (PBMCs). RNA-seq was performed using mRNAs of PBMCs pooled from three different mice of two or 20 months of age. Five hundred top and 500 bottom ranked genes were selected as HEGs and LEGs after transcriptomes were sorted by expression level in young T cells. Expression levels of old wild-type T cells relative to young were calculated using a log2 scale.
